# Physiological costs of chemical defence: repeated reflex bleeding weakens the immune system and postpones reproduction in a ladybird beetle

**DOI:** 10.1038/s41598-020-66157-9

**Published:** 2020-06-09

**Authors:** Michal Knapp, Michal Řeřicha, Dana Židlická

**Affiliations:** Department of Ecology, Faculty of Environmental Sciences, Czech University of Life Sciences Prague, Kamýcká 129, Prague, Suchdol 165 00 Czech Republic

**Keywords:** Behavioural ecology, Ecophysiology, Evolutionary ecology

## Abstract

In insects, external chemical defences, such as reflex bleeding, have been proved to be an efficient strategy against various predators. At the same time, significant costs of reflex bleeding can be expected because bled haemolymph is lost and all valuable components included have to be renewed. Interestingly, this issue has rarely been investigated for adult insects. In this study, we examined the immune and fitness costs of repeated reflex bleeding in adults of the invasive ladybird *Harmonia axyridis*, investigating several haemolymph parameters. Reflex bleeding induced twice a week for three weeks resulted in a significant reduction in haemocyte concentration, total protein content, and antimicrobial activity against *Micrococcus luteus*, and a marginally non-significant decrease in antimicrobial activity against *Escherichia coli*. Repeated reflex bleeding did not result in significant body mass reduction. Interestingly, resource limitation in the form of complete food absence did not significantly interact with reflex bleeding, even though starvation itself had a strong negative effect on all haemolymph parameters investigated and individual body mass. Daily reflex bleeding did not result in decreased fecundity of young ladybirds during the first 30 days of their adult life, but the start of ladybird reproduction was delayed by about two days. Moreover, ladybirds bleeding larger amounts of haemolymph started their reproduction significantly later. Overall, our results indicate that repeated reflex bleeding weakens a ladybird’s immune system and can increase their susceptibility to pathogens, but a ladybird’s reproductive potential remains almost unaffected, even by very intensive reflex bleeding.

## Introduction

Reflex bleeding (sometimes called “autohaemorrhaging”) is a well-known phenomenon which has fascinated biologists for centuries^1,2^. The release of haemolymph, when individuals are threatened or exposed to direct physical attack, is a common defensive behaviour in many insects with efficient toxins present in their bodies. Reflex bleeding behaviour has been described, for example, for Plecoptera, Orthoptera, and Hemiptera^3–5^, but it is most widespread in several beetle families, for example Meloidae, Erotylidae, Lampyridae, and Coccinellidae^6–9^. In addition, several insect species from other orders employ alternative mechanisms to externalise the toxins in their body fluids to predators, such as easy bleeding in sawfly larvae^10^ or regurgitation in lepidopterans, orthopterans, and others^11,12^. The efficiency of reflex bleeding behaviour in insects has been confirmed against various predators, including small mammals, birds, lizards, and arthropods^3,9,13–15^.

In general, all types of externalised chemical defence seem to be efficient defensive strategies against various predators^14^. Nevertheless, the externalisation of haemolymph can pose a significant physiological cost to insects because some of their haemolymph is lost and needs to be replenished. The limited existing evidence indicates that not only nutrients but also other valuable components of haemolymph are lost during reflex bleeding, e.g., haemocytes and alkaloids^16–18^. Reflex bleeding during the larval stage can result in reduced adult size or prolonged development time^9,19,20^. Species with external chemical defence can also be at a higher risk of parasitism by parasitoids^14^, probably due to a weakened immune system. Sometimes, reflex bleeding can be linked to genetic costs^21^. On the other hand, a recent study by Lee *et al*.^22^ showed no negative effect of repeated reflex bleeding on ladybird fitness (fecundity). However, the costs of reflex bleeding can also depend on the environmental context and physiological conditions of individuals^22^.

In this study, we investigated the effects of repeated reflex bleeding under various environmental contexts (resource availability) on basic haemolymph parameters and body mass change in the invasive ladybird *Harmonia axyridis* Pallas. We also measured the effects of frequent reflex bleeding during early adult life on ladybird reproductive performance (age at first reproduction and egg production). We hypothesized that all investigated haemolymph parameters and body mass are negatively affected by repeated reflex bleeding; in addition, we expected this effect to be strengthened by limited food availability because valuable components of haemolymph are lost during reflex bleeding (e.g., alkaloids, haemocytes and proteins with various functions)^16–18^. We also predicted that the reproductive performance of young adults is negatively affected by reflex bleeding more significantly than the reproductive performance of older adults (published in^22^); this is because the accumulation of biomass and investment in the immune system are very resource-intensive in the first days of adult life, as shown in our previous study describing the ontogeny of immune system in *H. axyridis*^23^.

## Results

### Experiment 1 – physiological parameters

**Figure 1:**
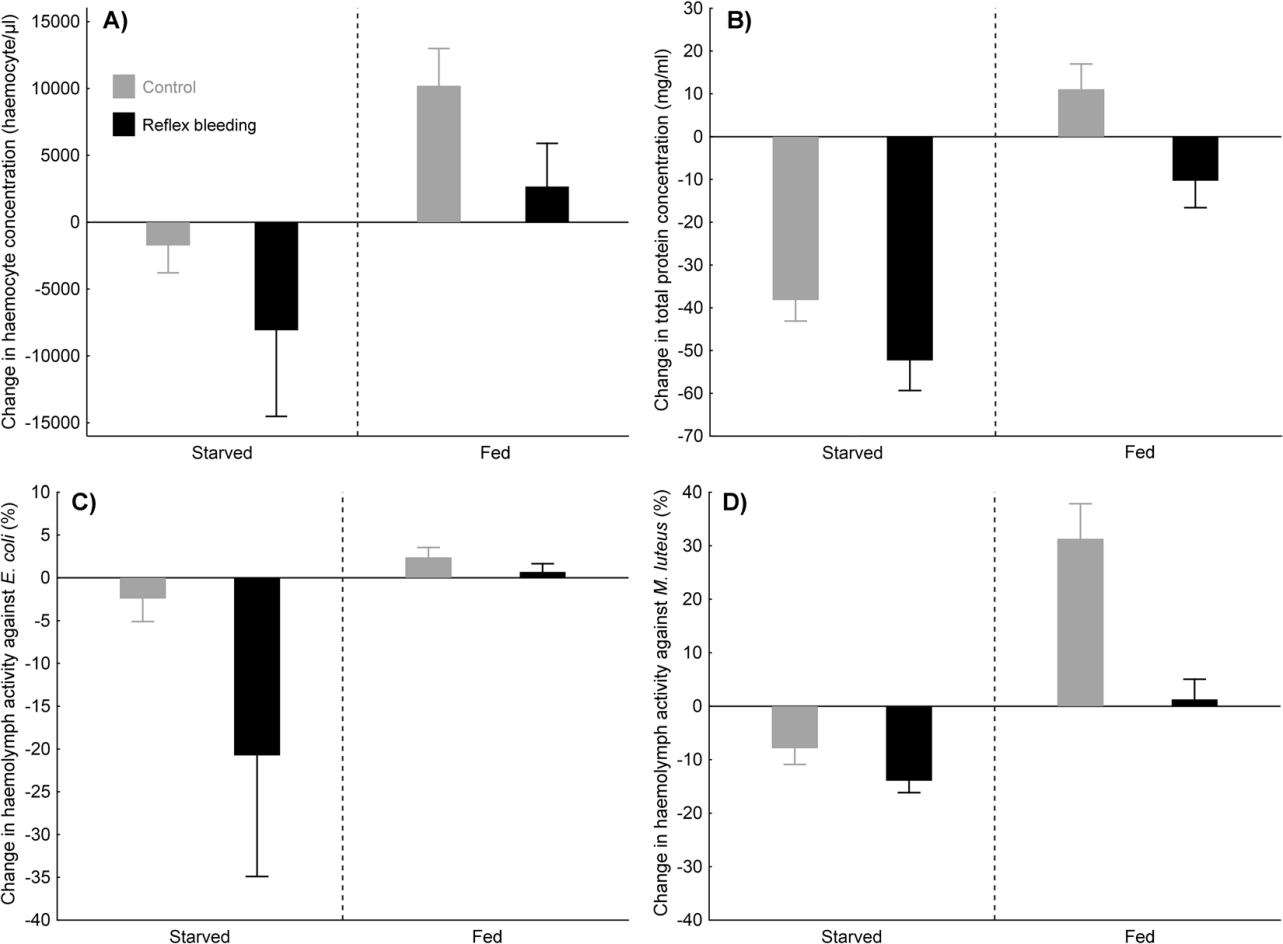
Effects of repeated reflex bleeding and starvation on haemocyte concentration, protein concentration, and anti-microbial activity in haemolymph in *Harmonia axyridis*. Reflex bleeding was induced twice a week during a three-week period. Mean change in these variables between the beginning and the termination of the experiment (three weeks later) and standard errors of means are shown: (**A**) haemocyte concentration; (**B**) total protein concentration; (**C**) antimicrobial activity against *Escherichia coli*; (**D**) antimicrobial activity against *Micrococcus luteus*.

Ladybird survival was negatively affected by starvation (Cox proportional hazards model: z = 3.76; P < 0.001; Fig. S1) but unaffected by repeated reflex bleeding and sex (Cox proportional hazards model: both P > 0.24). In contrast to survival, all investigated haemolymph parameters (i.e., haemocyte concentration, protein concentration and antimicrobial activity against *E. coli* and *M. luteus*) were negatively affected by repeated reflex bleeding (Fig. 1). This effect was significant for haemocyte concentration, protein concentration, and antimicrobial activity against *M. luteus* (ANOVA: P < 0.05 in all cases); it was marginally non-significant for antimicrobial activity against *E. coli* (ANOVA: P = 0.073; see Table 1, for details). Even stronger negative effects on all investigated haemolymph parameters resulted from ladybird starvation (Fig. 1; ANOVA: P < 0.02 in all cases). There was no significant interaction between feeding and reflex bleeding treatments affecting change in any of investigated physiological parameters; however, the interaction for antimicrobial activity against *E. coli* was only marginally non-significant (ANOVA: P = 0.056; see Table 1, for details). The statistical non-significance of this interaction could be caused by the low sample size available for the most stressful treatment combining limited resources (starvation) and repeated reflex bleeding.

**Table 1:** Effects of resource limitation (starvation), repeated reflex bleeding, and the interaction between starvation and reflex bleeding on body mass change, haemocyte concentration, total protein concentration and antimicrobial activity of haemolymph in *Harmonia axyridis*.

	Body mass (difference)		Haemocyte concentration (difference)		Total protein concentration (difference)		Antimicrobial activity - *E. coli* (difference)		Antimicrobial activity - *M. luteus* (difference)	
	F-value	P-value	F-value	P-value	F-value	P-value	F-value	P-value	F-value	P-value
Starvation	65.13	**<0.001**	6.21	**0.017**	34.72	**<0.001**	7.82	**0.012**	14.42	**<0.001**
Bleeding	0.19	0.67	4.14	**0.049**	9.24	**0.004**	3.64	0.073	16	**<0.001**
Starvation * Bleeding	0.52	0.48	0.03	0.87	0.18	0.67	4.18	0.056	0.81	0.37

Response variables represent changes in measured parameters between the beginning and the termination of the experiment (three weeks later). Detailed results of analyses of variance (ANOVA) are shown. Sex was included as covariate in all models (its significance differed between models and is not shown). Significant terms are highlighted in bold.

**Figure 2:**
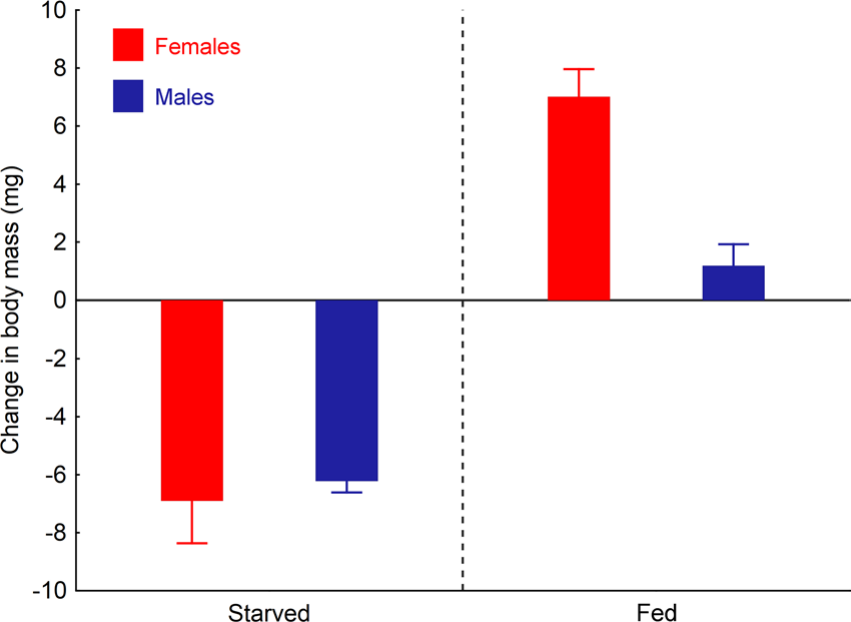
Effect of feeding regime (fully fed vs. starved individuals) and sex on body mass change of *Harmonia axyridis* beetles during the three-week period. There was no significant effect of repeated reflex bleeding (twice a week) on body mass change in our experimental beetles. Mean body mass changes (i.e., differences between the beginning and the termination of the experiment) and standard errors of means are shown.

Ladybird body mass change was significantly affected by the resource availability regime (ANOVA: F = 65.13; P < 0.001), with starved beetles losing body mass and fed beetles gaining mass during the experiment (Fig. 2). However, repeated reflex bleeding had no effect on ladybird body mass (ANOVA: F = 0.19; P = 0.67).

### Experiment 2 – early adult life reproductive performance

**Figure 3:**
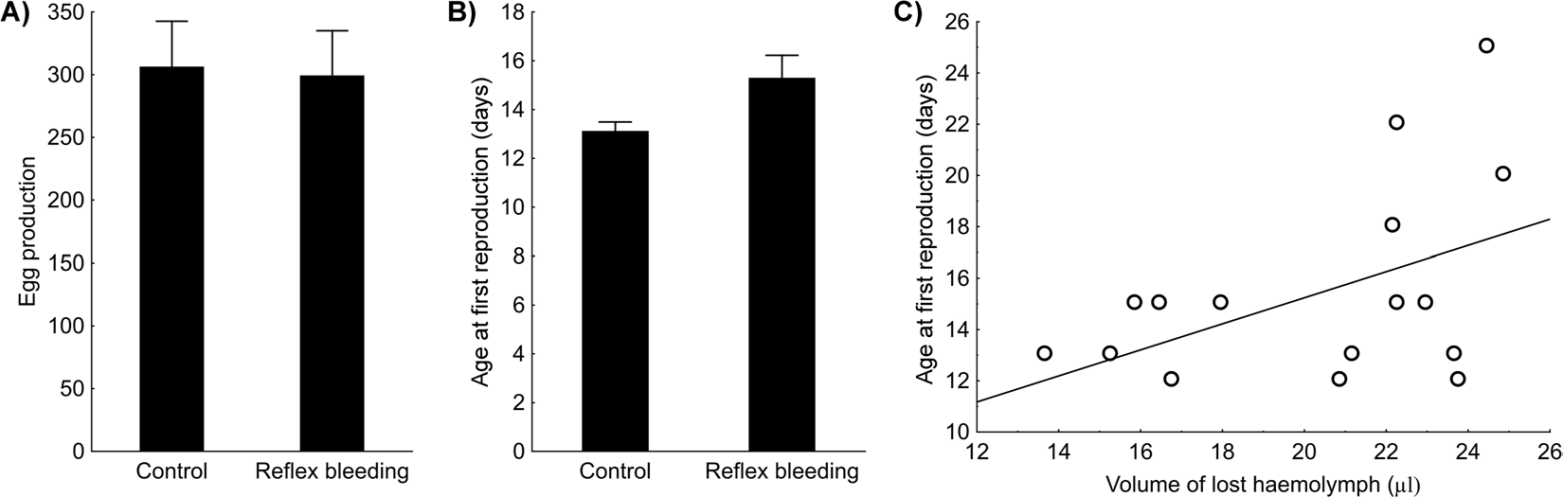
Effects of daily reflex bleeding on fecundity and age at first reproduction in *Harmonia axyridis* ladybirds in the first 30 days of their adult life. Mean egg production (per female per 30 days; panel (A) and mean age at first reproduction (in days; panel B), and standard error of mean are shown. The relationship between age at first reproduction and the volume of haemolymph lost for bled females is shown in panel C.

Total egg production during early adult life (first 30 days) was not affected by repeated reflex bleeding (Fig. 3A; GLM quasi-Poisson: F = 0.19, P = 0.89). However, repeated reflex bleeding significantly postponed age at first reproduction (Fig. 3B; ANOVA: F = 4.76, P = 0.037). In repeatedly bled females, the amount of lost haemolymph was not related to the number of eggs produced during first 30 days of their adult life (GLM quasi-poisson: F = 0.28, P = 0.60). On the other hand, females that lost larger amounts of haemolymph during repeated reflex bleeding started their reproduction significantly later (Fig. 3C; linear regression: F = 4.67, P = 0.047).

## Discussion

We demonstrated the existence of substantial immune and possible fitness costs linked to repeated reflex bleeding in adult ladybirds. Previous studies investigated physiological costs of reflex bleeding mainly during the larval stage^9^ or revealed only very limited fitness costs linked to repeated reflex bleeding in adult insects.

External chemical defences, such as reflex bleeding, are efficient against predators; however, the existence of its physiological costs can be indicated by the higher prevalence of parasitoids in species using external chemical defence than in species not using it^14^. Knapp *et al*.^18^ showed that, in ladybirds, the composition of reflex blood is exactly the same as of the haemolymph originating from body cavity. Thus, components valuable for immune system function, e.g., alkaloids, haemocytes or antimicrobial peptides, are lost and have to be renewed. Here we showed that haemocyte and protein concentrations are significantly reduced by reflex bleeding induced twice a week. Similarly, Holloway *et al*.^16^ and de Jong *et al*.^24^ showed that ladybirds in which reflex bleeding was induced just once a week were not able to produce alkaloids at a rate sufficient to compensate for their loss. The immune system of *H. axyridis* relies heavily on harmonine, an alkaloid with strong antimicrobial activity, as shown by the tight correlation between harmonine concentration and antimicrobial activity reported by Schmidtberg *et al*.^25^. A failure to compensate for the component loss would result in a weakened immune system, and thus resistance against pathogens and parasitoids would be compromised. It should be noted that haemocytes responsible for nodulation and encapsulation, antimicrobial peptides and alkaloids are the main cornerstones of the immune system in ladybirds^25–27^. In this study we provide the first direct evidence that repeated reflex bleeding really has detrimental effects on immune system functioning in *H. axyridis*. Antimicrobial activity against *M. luteus* was significantly reduced and antimicrobial activity against *E. coli* tended to be reduced after repeated reflex bleeding.

Negative effects of repeated reflex bleeding were weaker than the effects of feeding treatment (starvation) for all measured haemolymph parameters. Negative effects of limited nutrients on immune system function in insects have been reported previously^28^; however, our setting (long-term starvation after overwintering) was really detrimental for the beetles (some of them died during the experiment; for details see Fig. S1). Under such circumstances, the missing interactions between starvation and reflex bleeding revealed in our study are surprising because relatively lower allocation to immune system recovery can be expected under strongly limited resources. Our results could have been affected by high mortality in the most stressful treatment combining starvation and repeated reflex bleeding. Limited sample size probably resulted in low power of statistical tests and non-significant results, despite quite large differences visible in Fig. 1C for antimicrobial activity against *E. coli*. We recommend reinvestigating the interactions between resource limitation and reflex bleeding in a future study using various levels of resource limitation, ensuring high survival rates for at least some levels of resource limitation. Our extreme setting (i.e., long-term starvation) could also result in biased results due to selecting individuals in exceptional good physiological condition (those in worst condition died and could not be resampled at the end of the experiment).

In addition to components important for immune system functioning, reflex blood also contains nutrients that are needed for ladybird survival, growth, and reproduction^29–31^. Thus, a trade-off between this anti-predatory behaviour (i.e., frequent reflex bleeding) and allocation to growth and reproduction can be expected. Ladybirds, similarly to other temperate insects overwintering as adults, significantly deplete their energy reserves during winter and their body mass then increases rapidly during early spring^32,33^. Interestingly, we observed no effects of repeated reflex bleeding on body mass change in post-overwintering ladybirds, even under the starvation regime. A possible explanation is that body mass loss is compensated by increasing water content. Water content can be highly variable at intraspecific level in insects^34^ and we also observed that bled individuals were thirsty; in many cases they drank water immediately after a reflex bleeding event.

The existing literature also indicates missing or only limited trade-off between repeated reflex bleeding and reproductive investment in ladybirds. Published studies have reported mixed evidence for effects of reflex bleeding on egg fertility. Bayoumy *et al*.^35^ observed reduced fertility for eggs originating from bled females, whereas Lee *et al*.^22^ observed slightly increased fertility for eggs from bled females. Fecundity did not differ between daily bled and control beetles for three ladybird species during a 10-day experiment when older adults were bled^22^. Even food restriction did not result in limited fecundity for repeatedly bled beetles in the same experiment^22^. In our study, we focused on fitness consequences for young adults, which can be more resource limited than older ones. Our previous experiments confirmed that very young (1–8 days old) adults strongly allocate their energy into immune system and body tissues development^23^ (M. Knapp, unpublished data on body mass change with ageing in *H. axyridis*). Surprisingly, even this experimental setting did not result in reduced fecundity during the first 30 days of adult life. The only significant effect of daily reflex bleeding was observed for age at first reproduction, with bled females postponing their reproduction for about two days. This delay can represent a significant fitness cost for some insect species, but it is probably only of minor importance for ladybirds as their adults are long-lived with a long reproductive period^32^. The relationship between the timing of first reproduction and the volume of blood lost for individuals forced to reflex bleed was revealed. However, the causality remains unclear. Non-reproducing individuals can be more prone to stronger reflex bleeding (can afford it). On the other hand, haemolymph loss per se can postpone reproductive activities for heavily bleeding individuals. It is possible that direct fitness limitation observed for bled individuals is less important than transgenerational effects acting on the offspring of stressed ladybirds^35^; this issue deserves a  future attention.

In conclusion, our results indicate that repeated reflex bleeding weakens a ladybird’s immune system and can increase their susceptibility to pathogens, but a ladybird’s reproductive potential remains almost unaffected, even when young adults (for which strong energy limitation is expected) are exposed to repeated reflex bleeding events. The only significant effect of repeated reflex bleeding on ladybird reproduction was the delayed start of egg laying.

## Materials and Methods

### Study species

The harlequin ladybird, *Harmonia axyridis* (Pallas, 1773) investigated in this study is native to Eastern Asia, particularly to areas with a temperate and subtropical climate^36^. During recent decades it has been introduced to several European countries and to North America^37,38^. This aphidophagous species is known for its high voracity and high fecundity; its lifetime egg production can reach more than 5000 eggs^39^. High lifetime egg production is enabled by the long lifespan of adults, which commonly live for several months, but in extreme cases the lifespan of large ladybird’s can be up to two years^32^. Under field conditions, reflex bleeding can be frequently observed in *H. axyridis* and the willingness to eject haemolymph is relatively high when compared to other ladybird species under laboratory conditions (M. Knapp, unpublished data). The reflex blood of this species has the same composition as haemolymph sampled from the body cavity^18^.

### Experiment 1 – physiological parameters

*H. axyridis* adults were collected prior to their overwintering in October 2016 in the village of Konětopy, Czech Republic (coordinates: 50°16′26″N, 14°39′30″E). The ladybirds were overwintered in Prague-Suchdol under outdoor conditions in 1 litre glass jars in groups of about 50 individuals. In mid-April 2017, about 100 randomly selected survivors were individually accommodated in plastic Petri dishes 9 cm in diameter under standardized laboratory conditions (constant temperature 23 °C, long-day photoperiod 16 L:8D). Prior to the start of the experiment, all individuals were fed *ad libitum* with *Ephestia kuehniella* eggs and provided with water in modified Eppendorf tubes for one week. On the first day of the experiment, all individuals were weighed using a Sartorius balance with a precision of 10^−4^ g. Then, they were induce to reflex bleed by poking their legs with an entomological pin^18^. The volume of collected haemolymph varied between individuals, but only individuals who provide more than 0.5 µl were included in the experiment (40 males and 40 females were needed). The maximal volume of haemolymph collected was 2.2 µl, which equals about 5% of adult live mass. The ladybirds that produced enough haemolymph were evenly assigned to one of the four treatments: two feeding treatments (fed vs. starved) crossed with two reflex bleeding treatments (bled vs. control), that is 10 males and 10 females per each of the four treatments. Fed beetles were provided water and *E. kuehniella* eggs *ad libitum*, whereas starved beetles were only provided with water. Bled individuals were forced to reflex bleed twice a week. After the initial haemolymph sampling on the first day, five additional bleeding sessions were performed, meaning that bled individuals were stimulated to bleed six times in total. The volume of bled haemolymph varied between individuals: fed beetles bled on average 0.52 µl and starved beetles bled on average 0.44 µl. Control individuals were bled only on the first and the last day of the experiment. All individuals were reweighed on the last day immediately before terminal haemolymph sampling. If reflex bleeding did not provide a sufficient amount of haemolymph for the following physiological measurements (0.5 µl needed), the beetles were also subjected to puncture sampling of haemolymph at the end of the experiment. Puncture sampling consist of puncturing the metasternum of the ladybird with a sterilized entomological pin (diameter of 0.30 mm) and collecting the haemolymph that exuded from the puncture^18^. Survival of all individuals was recorded during reflex bleeding sessions, i.e., twice a week.

Haemolymph samples collected on the first and the last days of the experiment were immediately diluted (100x dilution) in anticoagulant buffer (62 mM NaCl, 100 mM glucose, 30 mM trisodium citrate, and 26 mM citric acid) and haemocytes were counted using a Bürker chamber under a Carl Zeiss Primo Star microscope^18^. The diluted haemolymph samples were frozen and, later, their protein content and antimicrobial activity against *Escherichia coli* were measured (see^23^, for details). Further, antimicrobial activity against *Micrococcus luteus* was measured using the radial diffusion method. Petri dishes were filled with a fresh nutritionally rich medium for the growth of bacteria (Lysogeny broth with agar: 10 g NaCl, 5 g yeast extract, 10 g tryptone and 15 g agar per litre of water), 100 µl of *M. luteus* culture (ca. 10^6^ cells/m^2^) was added per Petri dish and the content was homogenized. Later, holes were drilled into the agar and 5 µl of diluted haemolymph (100x dilution) was added. *M. luteus* bacteria were cultivated at 25 °C for 24 hours. The diameters of zones cleared around haemolymph samples were measured and calibrated using lysozyme standards.

To evaluate the effect of a particular treatment on change in a particular physiological parameter, the differences between the first and the last day values originating from the same individuals were analysed. For example, a change in haemocyte concentration was expressed as the haemocyte concentration on the last day minus their concentration on the first day. Positive values thus indicate an increase over the course of the experiment. Only individuals surviving until the end of the experiment and providing a sufficient amount of haemolymph (at least 0.5 µl) were included in our analyses of haemolymph parameters. All survivals were used for analysis of body mass changes (see Supplementary File Dataset S1). To statistically evaluate the effects of reflex bleeding and starvation, an analysis of variance (ANOVA) was used. All investigated response variables (change in body mass, haemocyte concentration, protein concentration, and antimicrobial activity against *E. coli* and *M. luteus*) met the assumptions needed, e.g., Gaussian distribution of errors. A separate model was fitted for each response variable. Feeding regime (fed vs. starved), reflex bleeding regime (bled vs. control), and the interaction term between feeding regime and reflex bleeding regime were used as independent variables in all models. Sex was used as a covariate in all models to control for possible differences between male and female responses. The significance of particular terms was tested using F-tests. Survival of experimental ladybirds was analysed using a Cox proportional hazards model with sex, starvation regime, and reflex bleeding regime as independent variables. All analyses were performed using R version 3.0.1^40^.

### Experiment 2 – early adult life reproductive performance

Five *H. axyridis* parental pairs were collected in Prague-Farkáň (coordinates: 50°3′39″N, 14°23′1″E) in May 2018, and brought back to the laboratory where they were fed and their eggs incubated. Their offspring were reared in Petri dishes under standardized laboratory conditions (constant temperature 23 °C, long-day photoperiod 16 L:8D, water and *E. kuehniella* eggs as food *ad libitum*) during their preimaginal development. Immediately after adult emergence, couples were established, consisting of male and female individuals that emerged on the same day but originated from different parental pairs (to exclude mating between closely related individuals). In total, forty couples were established and kept in Petri dishes. All individuals were provided with water and food (*E. kuehniella* eggs) *ad libitum* from the first day. On the second day, couples were assigned at random to two treatments: repeated reflex bleeding and control. Females in the repeated reflex bleeding treatment were subjected to reflex bleeding procedure daily (from the second day onwards), whereas females in the control treatment and all males were not stimulated to bleed at all. The ladybirds were provided with new food and water daily. At the same time, egg clutches were checked, and the number of eggs produced was counted. For females from the reflex bleeding treatment, the volume of bled haemolymph was measured using a glass microcapillary (Hirschmann, Germany) and a digital calliper. All investigated couples were followed for 30 days.

To analyse the effect of repeated reflex bleeding on the number of eggs produced in the first 30 days of their adult life, a generalised linear model (GLM) with a quasi-Poisson distribution of errors was fitted. The effect of repeated reflex bleeding on age at first reproduction (i.e., time to laying the first clutch, in days) was analysed using a one-way ANOVA. Treatment (repeated reflex bleeding vs. control) was the only independent variable in both models. In these analyses, only successfully mated females, i.e., those producing more than 10 eggs, were considered (see Supplementary File Dataset S1). To analyse the effect of haemolymph loss on egg production, a GLM with a quasi-Poisson distribution of errors was fitted. Total egg production was used as a response variable and total volume of haemolymph bled as an independent variable in this model. The relationship between age at first reproduction and total volume of haemolymph bled was analysed using a simple linear regression. The effects of haemolymph loss were only investigated for females from the reflex bleeding treatment. All analyses were performed using R version 3.0.1^40^.

## Supplementary information

Supplementary information.

Supplementary information 2.

## Data Availability

All the data produced during the study and analysed in this article are attached as Supplementary Material files (Dataset S1).
