# Supplementary material for: Physiological costs of chemical defence: repeated reflex bleeding weakens the immune system and postpones reproduction in a ladybird beetle
Source: Sci Rep. 2020 Jun 9;10:9266. doi: 10.1038/s41598-020-66157-9 (PMC7283328; doi:10.1038/s41598-020-66157-9)

Physiological costs of chemical defence: repeated reflex bleeding weakens the immune system and postpones reproduction in a ladybird beetle

MICHAL KNAPP[1*], MICHAL ŘEŘICHA[1], DANA ŽIDLICKÁ[1]

[1] Department of Ecology, Faculty of Environmental Sciences, Czech University of Life Sciences Prague, Kamýcká 129, Prague - Suchdol, 165 00, Czech Republic

* Correspondence:
Michal Knapp, Department of Ecology, Faculty of Environmental Sciences, Czech University of Life Sciences Prague, Kamýcká 129, Praha – Suchdol, 165 00, Czech Republic. Tel. +420 22438 3853; e-mail: knapp@fzp.czu.cz
ORCID: 0000-0003-4487-7317

Figure S1:

Effect of feeding regime (fully fed vs. starved individuals) on survival of experimental *Harmonia axyridis* beetles during the 18-day experimental period. Time points correspond to following sampling days: T0 = 4th day, T1 = 7th day, T2 = 11th day, T3 = 14th day, T4 = 18th day and T5 = 21st day. There were no significant effects of reflex bleeding treatment and sex on survival of experimental individuals.

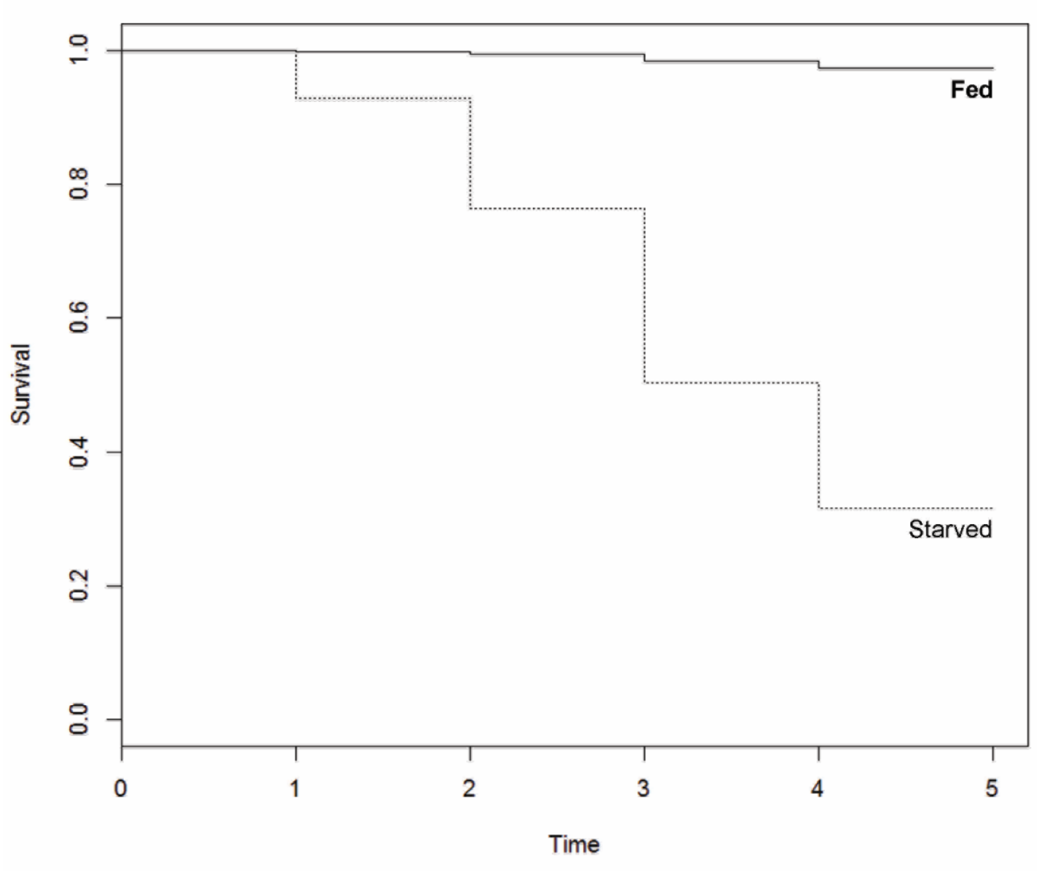

Supplement: Supplementary file 1 — Supplementary information. [file 41598_2020_66157_MOESM1_ESM.pdf]
